# Gonadoblastoma and Papillary Tubal Hyperplasia in Ovotesticular Disorder of Sexual Development

**DOI:** 10.4274/jcrpe.2705

**Published:** 2016-09-01

**Authors:** Enver Şimşek, Çiğdem Binay, Meliha Demiral, Baran Tokar, Sare Kabukçuoğlu, Melek Üstün

**Affiliations:** 1 Osmangazi University Faculty of Medicine, Departments of Pediatric Endocrinology, Eskişehir, Turkey; 2 Osmangazi University Faculty of Medicine, Departments of Pediatric Surgery, Eskişehir, Turkey; 3 Osmangazi University Faculty of Medicine, Departments of Pathology, Eskişehir, Turkey

**Keywords:** Ovotestes, sex-determining region on the Y chromosome, gonadoblastoma, dysgerminoma, tubal hyperplasia

## Abstract

Ovotesticular disorder of sexual development (DSD), formerly known as true hermaphroditism, is a rare form of DSD in which both testicular and ovarian tissues are present in the same individual either in a single gonad (ovotestis) or in opposite gonads with a testis and an ovary on each side. The diagnosis of ovotesticular DSD is based solely on the presence of ovarian and testicular tissue in the gonad and not on the characteristics of the internal and external genitalia, even if ambiguous. Herein, we report two patients with ovotesticular DSD-one presenting with ambiguous genitalia on the third day after birth and the other with short stature and primary amenorrhea in adolescence. Clinical and histopathological investigation revealed a sex-determining region on the Y chromosome (SRY)-positive 46,XX karyotype and bilateral ovotestes in case 1 and a 46,XY karyotype with hypergonadotropic hypogonadism and a streak gonad in one ovotestis with dysgerminoma, gonadoblastoma, and papillary tubal hyperplasia in the contralateral ovotestis in case 2. Laparoscopic examination and gonadal biopsy for histopathological diagnosis remain the cornerstones for a diagnosis of ovotesticular DSD. Moreover, SRY positivity in a 46,XX patient, a 46,XY karyotype, an intra-abdominal gonad, and the age of patient at the time of diagnosis are predictive risk factors for the development of gonadoblastoma and/or dysgerminoma in ovotesticular DSD.

**WHAT IS ALREADY KNOWN ON THIS TOPIC?**

Ovotesticular disorder of sexual development (DSD) is a rare form of DSD. Delayed diagnosis and gonadectomy are the main reasons for development of gonadal malignancy in case of ovotesticular DSD.

**WHAT THIS STUDY ADDS?**

We report the first case of papillary tubal hyperplasia associated with gonadoblastoma and dysgerminoma in a case of ovotesticular DSD.

## INTRODUCTION

Ovotesticular disorder of sexual development (DSD) can be diagnosed based only on histological criteria by detection of presence of both ovarian (containing follicles) and testicular tissue in the same gonad (ovotestis) or by the morphological appearance of the contralateral gonad. Approximately 60% of such patients have a 46,XX karyotype, 33% have a 46,XX/46,XY sex chromosome mosaicism, while the remaining 7% of patients have a 46,XY karyotype (1). Rarely, a sex-determining region on the Y chromosome (SRY) mutation may be identified in 46,XY ovotesticular DSD (2). On the other hand, SRY is present in only 10% of ovotesticular DSD cases with a 46,XX karyotype (3,4,5,6). The testes or testicular components of the ovotestes in ovotesticular DSD are likely to be dysgenetic, and gonadal tumors with a potential for malignancy occur in 2.6% of all cases (7). Dysgerminomas, gonadoblastomas, seminomas, and yolk sac carcinomas have been reported in ovotesticular DSD (8,9). Here, additional dysgerminoma and gonadoblastoma in a patient with 46,XY ovotesticular DSD and SRY positivity in a patient with 46,XX ovotesticular DSD are reported.

In this study, we report two cases of ovotesticular DSD for three reasons. Firstly, we wished to emphasize that cases of ovotesticular DSD may present at any age. Early diagnosis and prophylactic gonadectomy of ovotestesticular gonads can prevent the diagnostic delay for gonadoblastoma and/or dysgerminoma. Secondly, we wanted to show that pelvic ultrasound examination compared with laparoscopic examination has a relatively low sensitivity and specificity for establishing the presence or absence of gonads and Müllerian or Wolffian derivatives in DSD patients. Finally, to the best of our knowledge, we report the first case of papillary tubal hyperplasia (PTH) associated with gonadoblastoma and dysgerminoma in a case of ovotesticular DSD.

## CASE REPORTS

### Case 1

**Figure 1:**
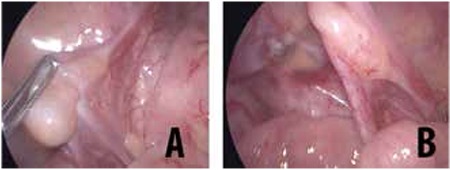
Appearance of external genitalia in case 1. There was a phallus 2.3-cm in length, bifid labioscrotal folds, absence of genital skin pigmentation (B), incomplete labioscrotal fusion (between Prader stages II and III), ventral opening urethra, and chordae (C)

**Figure 2:**
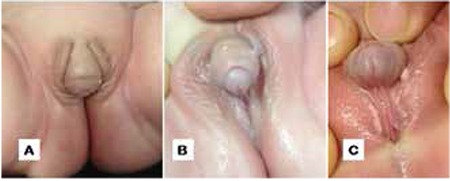
In case 1, appearance of the left (A) and right (B) gonads upon laparoscopic examination. The gonads were located in deep retrocolic areas

**Figure 3:**
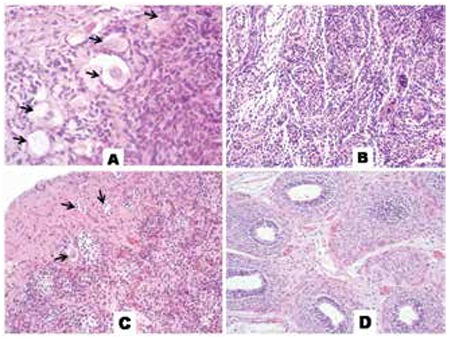
Histological examination of gonadal biopsy samples for case 1 revealed features of ovotestes which are characterised by ovarian stroma (H&E, x400) (A), and disorganised seminiferous tubules (H&E, x200) (B), right gonad. The ovarian follicles are seen on the left (arrows) surrounded by numerous small seminiferous tubules with immature Sertoli cells (H&E, x20). (C) The epididymis is lined by tall columnar cells, left side (H&E, x20) (D), left gonad

The first patient was referred to our institution on the third day after birth due to ambiguous genitalia. The patient was born at term after a normal pregnancy with a birth weight of 3250 g, a body length of 51 cm, and a 35.5-cm head circumference and was the first child of unrelated parents. Except for this case, the medical history of the family was normal. Upon physical examination, the patient was found to have ambiguous genitalia (Figure 1) including a phallus with a length of 2.3 cm, bifid labioscrotal folds, incomplete labioscrotal fusion (between Prader stages II and III), a ventral opening of the urethra and chordae. The gonads were non-palpable bilaterally. In addition, there was no genital skin pigmentation. The initial workup was negative for congenital adrenal hyperplasia (CAH) and included measurements of 17-hydroxyprogesterone (85 ng/dL; normal 7-77 ng/dL), 11-deoxycortisol (27 ng/dL; normal 13-147 ng/dL), Δ4 androstenedione (62 ng/dL, normal 20-290 ng/dL), and dehydroepiandrosterone sulfate (59.7 μg/dL, normal 15-120 μg/dL). Additional hormone analysis revealed a follicle-stimulating hormone (FSH) level of 0.8 mIU/mL (normal, none to 5.5 mIU/mL), a luteinising hormone (LH) level of 0.1 mIU/mL (normal 0.02-0.3 mIU/mL), a total testosterone (T) level of 205 ng/dL (normal 20-187 ng/dL), an estradiol (E2) level of 26 pg/mL, and an anti-Müllerian hormone (AMH) level of 10 ng/mL (normal reference values for <1-year-old males 101.9-262.0 ng/mL, for <14-year-old females 0.3-11.2 ng/mL). Chromosomal analysis and fluorescence in situ hybridisation (FISH) of SRY revealed a SRY-positive 46,XX karyotype. A human chorionic gonadotropin (hCG; Pregnyl^®^ 1500 U) test was performed to investigate presence of functional testicular tissue in the abdomen and testosterone synthesis defects. The test protocol included collection of a baseline blood sample (before the first injection of hCG between 8:00 and 9:00 a.m. on day 1) and collection of a second blood sample 24 h after the third injection of hCG to measure total T, dihydrotestosterone (DHT), and Δ4 androstenedione levels. Immediately following collection of the baseline blood sample, hCG (1500 U/1.73 m2/dose) was administered intramuscularly for three days. Basal total T was 192 ng/dL; DHT, 26 ng/dL (normal, 16-79 ng/dL); Δ4 androstenedione, 38 ng/dL (normal, 5-45 ng/dL); and T/DHT ratio 7.4 (normal, 11.1±4). Following three injections of hCG, T was 603 ng/dL; DHT, 57 ng/dL; Δ4 androstenedione, 57 ng/dL; and T/DHT ratio 10,6 (normal <20). The hCG test confirmed the presence of functional testes or testicular tissues in the undescended gonads. Pelvic ultrasonography (US) showed a bicornuate uterus, tuba uterina, and round ligaments; neither gonad could be identified in pelvic areas or inguinal channels. Using laparoscopic examination, Müllerian remnants were identified and consisted of a gonad, a fallopian tube adjacent to the gonad with a bilateral fimbriated end (Figure 2), and a bicornuate uterus. The gonads were deeply located in retrocolic areas. Bilateral longitudinal wedge gonadal biopsies were performed to complete the SRY-positive 46,XX DSD evaluation. Histopathological examination of gonadal biopsies demonstrated features of bilateral ovotestes (Figure 3). The parents were informed that patients with SRY-positive 46,XX DSD and ovotesticular gonadal structures have a high risk of the development of malignant gonadal tumours in the future. At age 6 months, the parents decided to raise the child as a female and gave permission for prophylactic gonadectomy. Bilateral gonadectomy was performed using the laparoscopic method.

### Case 2

**Figure 4:**
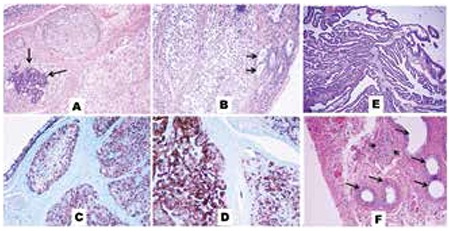
Histological examination of gonadoblastoma with superimposed dysgerminoma in the left gonad (A-D) and streak right gonad (E, F). (A) A focus calcification (arrows) lies in a dysgerminoma nest and tumour nests were encapsulated by immature granulose/Sertoli cells (middle upper) and were progressing to gonadoblastoma (H&E, x200). (B) Sertoli cells (arrows) and dysgerminoma nests progressing to gonadoblastoma (H&E, x400). Immunohistochemically, dysgerminoma cells showed reactivity with placental alkaline phosphatase (PLAP) (PLAP x400) (C) and c-kit (CD117) (CD117 x400) (D). (E) The right streak gonad showed polypoid and papillary hyperplasia of the tubular epithelium. Multiple small papillae floating in the tubal lumen (H&E, x200). (F) Leydig cell remnants (arrowheads) and epididymis (arrows) (H&E, x200)

**Figure 5:**
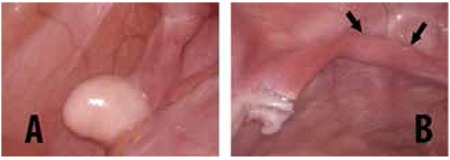
Laparoscopic images of the inner genital structures of case 2. (A) Tubular structure arising from the uterus ended with the left gonad. (B) Endoclips following a left gonadectomy and rudimentary uterus (arrows)

A 15-year-old female patient presented with absence of pubertal development, primary amenorrhoea and parental concern regarding severe growth retardation. She was the first child of non-consanguineous parents. The medical history of the family was noncontributory. The patient was born at term with a length of 50 cm and a weight of 3450 g; the neonatal period and infancy were uneventful. At physical examination, she was prepubertal with Tanner stage I breast development and stage III pubic hair development. Her height was 149 cm [-2.1 standard deviation (SD)] which is below the third percentile (mid-parental height; between the 10th and 25th percentiles), and her weight was 48 kg (-0.72 SD), i.e. between the 10th and 25th percentiles. Physical examination revealed a normal female external genitalia phenotype. Further physical examination was unremarkable although her bone age was delayed by 3.5 years (Greulich and Pyle method). A baseline blood screen (urea, electrolytes, calcium, phosphate, liver enzymes, and full blood count), screening for thyroid function, and screening for coeliac disease antibodies revealed normal values. Hormone assays revealed low oestradiol (<10 pg/mL; normal 21-85 pg/mL), high FSH (73.7 mIU/mL; normal 1.8-11.2 mIU/mL), and high LH (33 mIU/mL; normal 1.5-9.0 mIU/mL) levels, indicating hypergonadotropic hypogonadism. Pelvic US revealed a small uterus 4.7 cmx1.3 mm in diameter and hypoplastic gonads (left pelvic gonad: 15 x 12 mm; right pelvic streak gonad: 7x6 mm). Due to the presence of a hypoplastic Müllerian structure, streak gonad, short stature, and primary amenorrhoea in this patient with a female phenotype and hypergonadotropic hypogonadism, a tentative diagnosis of Turner syndrome was made. However, the characteristic physical stigmata of Turner syndrome were not present. Chromosomal analysis and FISH of SRY showed an SRY-positive 46,XY karyotype. These findings indicate that gonadal dysgenesis should be considered in this differential diagnosis. Due to the fact that an SRY-positive 46,XY genotype and rudimentary gonads are associated with a high risk of malignancy, the parents and patient were informed of the malignancy risk of dysgenetic gonads. A bilateral gonadectomy was carried out by the laparoscopic method. Histopathological examination of the excised left gonad revealed features of an ovotestis with both ovarian and testicular tissues present in addition to a gonadoblastoma on the base of dysgerminomas (Figure 4). Histopathological examination of the right rudimentary gonad revealed streak gonad parenchyma with epididymis and papillary tuba hyperplasia (PTH; Figure 4D). Laparoscopic examination revealed a left tubular structure arising from the rudimentary uterus that ended with the left gonad (Figure 5), and a streak right gonad.

## DISCUSSION

Ovotesticular DSD is associated with heterogeneous clinical, genetic, and pathological spectra. Patients often present in the neonatal period with ambiguous genitalia, as in case 1. However, patients may present with a normal female phenotype in adolescence or adult life, as in case 2. Investigation of the aetiology of DSD should be managed in a tertiary centre by a multidisciplinary team that includes a geneticist, a pediatric urologist or pediatric surgeon, and a pediatric endocrinologist.

Many excellent algorithms for the investigation of the aetiology of DSD are available. Palpable or non-palpable gonads, genital skin pigmentation, electrolytes, patient age at presentation, and family history are key clinical issues at initial evaluation. One of the current cases presented with ambiguous genitalia, non-palpable gonads, no genital skin pigmentation, and normal electrolytes. Many of these findings are inconsistent with a diagnosis of CAH, however, clinicians should rule out the diagnosis of CAH with an additional hormonal analysis. Here, the diagnosis of CAH was excluded by basal hormonal analysis. The second case presented with a normal female phenotype at the age of 15 years with short stature, primary amenorrhoea, and hypergonadotropic hypogonadism. In both cases, the next stage of diagnostic investigation consisted of SRY and chromosomal analyses. Case 1 exhibited an SRY-positive 46,XX karyotype and case 2 exhibited a SRY-positive 46,XY karyotype. These findings ruled out Turner syndrome in case 2. At this point, a diagnosis of gonadal dysgenesis as the aetiology of the SRY-positive 46,XX and 46,XY DSD cases was very close. The last step was the imaging of Müllerian and/or Wolffian derivatives, undescended gonads, and gonadal biopsy and/or gonadectomy for histopathological diagnosis.

The uterus and ovaries are relatively easy to find during the neonatal period using pelvic US, since these structures are prominent under the influence of maternal hormones (10). On the other hand, many studies discourage the use of pelvic US as the primary modality for establishing the presence or absence of gonads. Cohen et al (11) reported that only one ovary is identified in 40% of typical patients and none in 16%. Steven et al (12) was the first to assess the reliability of pelvic US in the evaluation of Müllerian derivatives in children with DSD and reported significant limitations, because pelvic US has only 54% sensitivity and 50% specificity. A recent meta-analysis of 12 studies (591 testes) indicated that pelvic US has an overall sensitivity and specificity of 44% and 95%, respectively (13). In the present study, neither gonad could be identified by pelvic US in one of the two cases; however, laparoscopic investigation revealed both gonads located deep in retrocolic areas. An additional advantage of laparoscopic investigation is the option to obtain a gonadal biopsy, as in case 1, or to perform a gonadectomy during the laparoscopic examination, as in case 2.

Informative predictive parameters for the development of gonadal type 2 germ cell tumours are the anatomical position of the gonad, gonadal differentiation, and the presence of a specific part of the Y chromosome (14). The majority of patients with ovotesticular DSD have 46,XX karyotypes (60%), while the remaining patients have 46,XY (12%) or mosaic karyotypes (28%) (1,15). SRY is present in only 10% of 46,XX ovotesticular DSD patients (3,4,5,6). The presence of SRY in a patient with 46,XX ovotesticular DSD strongly suggests that the ovotesticular gonads carry a high potential for malignancy, especially dysgerminoma (7,14). On the other hand, gonadoblastomas have been reported to be rarely present in patients who have a normal 46,XX karyotype (16).

The gonadoblastoma locus (GBY) is the only oncogenic locus on the human Y chromosome. It is postulated to serve a normal function in the testis but could exert oncogenic effects in dysgenetic gonads of individuals with DSD (17). The development of a gonadoblastoma is dependent on the presence of part of the Y chromosome, known as the GBY region (15). One of the putative candidate genes for the involvement of this region is the testis-specific protein Y (TSPY) on the Y chromosome (18). It serves normal functions in male stem germ cell proliferation and differentiation (17). Ectopic expression and actions of TSPY gene in incompatible germ cells, such as those in dysgenetic or ovarian environments and dysfunctional testis, such as ovotesticular gonad, disrupt the normal cell cycle regulation and predispose the host cells to tumorigenesis. The encoded protein is found to be highly expressed in carcinoma in situ and gonadoblastoma (16). Ectopic expression of SRY, as a part of the Y chromosome in case 1 diagnosed with 46, XX ovotesticular DSD is the main risk factor for the development of gonadoblastoma. On the other hand, the second patient, diagnosed as a case of 46, XY DSD, has two well-known risk factors for the development of gonadoblastoma. One of them is presence of ovotesticular gonads and the second - the intra-abdominal localization of these gonads with testicular components for 15 years.

Histological examination of gonad biopsies in case 1 revealed bilateral ovotestes. Histological examination of excised gonads in case 2 revealed features of an ovotestis with a gonadoblastoma and dysgerminomas in the left gonad and streak gonad parenchyma and PTH in the right gonad. Gonadoblastoma and/or dysgerminomas in cases of ovotesticular DSD are well-known findings, however, PTH is a novel finding in ovotesticular DSD. Originally described by Kurman et al (19), PTH was identified in 20 (91%) of 22 patients with ovarian non-invasive low-grade serous tumours. According to this description, PTH is the most-advanced stage of tubal hyperplasia and characterised by tubal proliferations that exhibit papillary tufting and detached clusters of bland epithelium. These clusters of epithelial cells and small papillae are found floating in the lumen or protruding from the tubal mucosa into the lumen. The authors concluded that PTH is likely the precursor lesion and the small papillae and clusters of cells from the fallopian tubes implant on ovarian and peritoneal surfaces resulting in generation of low-grade serous tumours. Robey and Silva (20) found PTH in 68.7% of patients with ovarian serous borderline tumours. To our knowledge, this is the first report of PTH associated with ovotesticular DSD. PTH may be another tumour precursor in ovotesticular DSD.

In conclusion, in patients with gonadal dysgenesis or ovotesticular DSD, laparoscopic examination is the most sensitive and specific imaging modality for the evaluation of Müllerian derivatives and undescended gonads. This procedure has additional advantages, such as the potential to perform a gonadal biopsy for histopathological diagnosis or a gonadectomy to prevent or treat malignancy development. The presence or absence of SRY should be routinely investigated in patients with DSD because if a patient with ovotesticular DSD has Y-chromosome or SRY positivity, ovotestes or streak gonads should be excised before the development of gonadal malignancy. Furthermore, if PTH is identified following a histopathological examination, rudimentary Müllerian structures should also be excised completely to prevent gonadal or peritoneal serous tumours.

## Ethics

Informed Consent: Informed consent was obtained from the patient’s parents to participate in the study.

Peer-review: Externally peer-reviewed.
